# The Sibs Program: A Structured Peer-Mentorship Program to Reduce Burnout for First-Year Medical Students

**DOI:** 10.15694/mep.2020.000287.1

**Published:** 2020-12-23

**Authors:** Gabriel Arenas, Gregory Brisson

**Affiliations:** 1 The University of Chicago Pritzker School of Medicine; 2 Northwestern University Feinberg School of Medicine

**Keywords:** Peer-mentorship, medical student, medical school, anxiety, work-life balance.

## Abstract

This article was migrated. The article was marked as recommended.

First-year medical students are at risk for burnout despite resources available to help them manage stress. In 2015, a structured peer-mentorship program was created at our institution for incoming medical students (M1s) by second-year medical students (M2s) with a goal of reducing risk factors for burnout; a secondary goal was to improve the mentoring skills of M2s. Over the course of the year, we surveyed M1s about their anxiety, prioritization skills, and work-life balance; M2s from the previous year’s unstructured peer-mentorship program served as a control group. 164 M1s and 164 M2s participated in this program. Among M1s, a structured peer-mentorship program significantly reduced anxiety levels (p=<0.01), improved prioritization skills (p=<0.01), and facilitated greater awareness of the importance of striving to maintain work-life balance (p=<0.01). M2s felt neutral-to-agreeable in their ability to provide guidance, refer students for help, and remain invested in their mentees. A structured peer-mentorship program, therefore, may reduce anxiety, improve prioritization skills, and emphasize the importance of work-life balance among M1s, elements that have been associated with reduced rates of burnout. Furthermore, this program can augment the professional development of M2s by motivating them to maintain longitudinal mentoring relationships with underclassmen.

## Introduction

The medical school environment poses unique challenges to students regarding work-life balance, career exploration, and acquiring clinical skills and knowledge ( Dyrbye, Thomas and Shanafelt, 2005). These challenges are particularly difficult for first-year students (M1s) ( Dyrbye, Shanafelt, *et al.*, 2017; Hill, Goicochea and Merlo, 2018). Many members of the medical school community, including faculty, administrative personnel, and senior medical students facilitate the transition into medical school. In addition, various student groups and online resources assist M1s in adjusting to their new environment ( Karp and Levine, 2018). These elements are widely available at U.S. medical schools. However, students remain at risk for burnout, a state of physical and mental exhaustion strongly influenced by work responsibilities and professional duties ( Ishak *et al.*, 2013).

A study of nearly 4,300 students at seven medical schools found a 50% burnout rate, suggesting that many students could benefit from additional support ( Dyrbye, Thomas, Massie, *et al.*, 2008). A subsequent study identified a correlation between medical student burnout and alcohol abuse, suggesting that dysfunctional coping behaviors may arise in the setting of burnout ( Jackson *et al.*, 2016). As a result, medical schools have recognized the importance of improving the medical school environment to minimize burnout ( Dyrbye, Thomas and Shanafelt, 2005).

Burnout can be attributed to several factors, including increased anxiety, poor prioritization skills, and work-life imbalance ( Weber and Jackel-Reinhard, 2000; Dunn *et al.*, 2008; Drummond, 2015; van Dam, 2016; Baker and Sen, 2016). Anecdotal reports suggest that many schools use peer-mentoring programs to reduce and prevent burnout in students ( Andre *et al.*, 2017; Hogan *et al.*, 2017). However, the value of a structured peer-mentorship program has not been studied.

Our institution had a longstanding peer-mentoring program (called the “Sibs Program”) designed to facilitate interactions between second-year medical students (M2s or “big sibs”) and first-year medical students (M1s or “little sibs”). This program was unstructured, denoting no formal requirement for sibs to meet or direct responsibility for the big sib to mentor his or her little sib. Feedback about the program was mixed. While some of the sib pairings resulted in longitudinal mentoring relationships, many sib relationships did not persist beyond the first month of school. This observation could be attributed in part to increased academic demands from M2s and reluctance of M1s to seek advice from their big sib.

In 2015, we implemented a structured peer-mentorship program designed to promote greater interaction between sibs throughout the academic year and enhance mentoring. Our goal was to improve the transition to medical school for M1s by reducing burnout risk as assessed through their anxiety, prioritization skills, and importance for maintaining a work-life balance. A secondary goal was to improve the mentoring skills of M2s.

## Methods

Program Design

Our peer mentorship program was structured as follows: incoming M1 students were randomly assigned into one of four societies by the medical school administration. A society represents a community of students across all years of training intended to facilitate interclass mentoring sessions and social events. Student leaders in the societies paired each M1 with an M2 based on similar undergraduate institutions and degree backgrounds. Student participation was required unless exempted by their society’s faculty mentor. Throughout the academic year, M2s received emails every four to six weeks containing talking points that prompted them to interact with their little sib as shown in Table 1.

**Table 1.** Modules within the M1 Curriculum and Associated Talking Points

M1 Module	Talking Point
Foundations 1	Setting expectations for mentoring and starting medical school
Foundations 2	Selecting a research mentor, project, and dual degree discussions
Foundations 3	Preparing for an objective skills clinical exam (OSCE)
Cardiovascular + Blood	Getting involved with and transitioning into student groups, research, and community service
Pulmonary	Exploring different study strategies and academic resources
Renal	Exploring different specialties
Musculoskeletal + Dermatology	Reflecting on the year and changes to make in the upcoming year

Each module was paired with a talking point focused on predictable challenges and career guidance between siblings. These talking points were included in the email prompts received by the M2s to discuss with their little sib. These talking points were formulated by an ad hoc committee of students and faculty focused on addressing times of predictable stress in the M1 academic year. Students were encouraged to interact in person. Other alternative modes of acceptable communication between siblings included texting, calling, emailing, FaceTiming, and Skyping.

This student-directed initiative was designed to encourage M2s to remain engaged with their little sib and provide a greater number of M1s with the opportunity to have a meaningful mentoring relationship with an upperclassman. Of note, M2s did not receive any mentoring training prior to the initiation of the structured peer-mentorship program.

Study Design and Data Analysis

This was a prospective longitudinal study of M1s and M2s at Northwestern University Feinberg School of Medicine (FSM) in Chicago, IL, USA. Incoming M1s were the experimental group, participating in the new structured peer-mentorship program; M2s, having participated in an unstructured peer-mentorship program as M1s the year before, served as the control group.

The study population included all M1s and M2s enrolled at FSM with a big or little sib, respectively. Students were not included if they did not participate in the peer-mentorship program. Approval of this study was exempted by the Northwestern University Institutional Review Board.

During the academic year, M1s and M2s were evaluated with voluntary, anonymous Google surveys using a 5-point Likert scale (1 - strongly disagree; 5 - strongly agree) according to the timeline provided in Table 2 (surveys A, B, C, and D). An ad hoc committee of students and faculty developed these surveys and questions for this study specifically. Validated questions or surveys were not included as part of our study.

**Table 2.** M1 and M2 Structured Peer Mentorship Program Evaluation Timeline

Year	Block 1					Block 2				
	Aug.	Sept.	Oct.	Nov.	Dec.	Jan.	Feb.	Mar.	Apr.	May
	FDN 1	FDN 1	FDN 2	FDN 3	FDN 3	CVB	PULM	RENAL	MSK/Derm	MSK/Derm
**M1**	**A**				**E**					**E, C**
**M2**	**B**									**D**

Survey	Details	Response Rate
Survey A	M1 initial assessment of starting medical school	N = 128 (78%)
Survey B	M2 opinion on previous mentorship model	N = 105 (64%)
Survey C	M1 end of year transitioning into medical school	N = 85 (52%)
Survey D	M2 growth as mentors	N = 61 (37%)
Survey E	M1 assessment of M2 big sibs	Block 1: N = 90 (55%); Block 2: N = 79 (48%)

**Each letter in the timeline in Table 2 corresponds with the lettered survey and details listed. The length of this timeline is one academic school year. Under each month, an abbreviated name for each module is represented (FDN 1: Foundations 1; FDN 2: Foundations 2; FDN 3: Foundations 3; CVB: Cardiovascular and Blood; PULM: Pulmonary; RENAL: Renal; MSK/Derm: Musculoskeletal and Dermatology). Response rates are noted for each survey administered.** The M1 year was split into two blocks in order to achieve a midpoint and final assessment of the program by M1s. During these program assessments, we surveyed M1s on their number of mentoring discussions between siblings, their reasoning for fewer than 3 mentoring discussions between siblings per block, and their descriptions of any conflicts between siblings (survey E). A mentoring discussion was defined as an in-person meeting between siblings. If sibling pairs were unable to meet in person, other communication methods were used but were not surveyed. Two or fewer mentoring discussions per block represented less than optimal participation in the structured peer-mentorship program.

Prior to the initiation of the new structured peer-mentorship program, we surveyed M2s about their experience as M1s with the unstructured peer-mentoring program, focusing on anxiety, prioritization skills, and work-life balance (survey B). After one year in the structured program, we surveyed M2s on their mentoring skills by assessing their comfort in giving advice and guidance, motivation to help their sib, capabilities with referring sibs to additional resources, and identification of areas for growth in their little sib.

At the beginning of the academic year, we assessed M1s to measure their anxiety and comfort level after being assigned to a big sib (survey A). At the end of the year, we surveyed them about their anxiety, prioritization skills, and importance of work-life balance (survey C).

Data from survey B was compared with the results from survey C using an unpaired student’s t-test where *p* < 0.05 was determined to be statistically significant. The total number of responses were recorded with the mean, median, and standard deviation calculated for each question asked in all surveys.

## Results/Analysis

A total of 164 M1s and 164 M2s participated in the structured peer-mentorship program. Only one M2 was excluded from the sibling program.

The duration of the unstructured program was approximately 10 months (August 2014 - May 2015) compared to the structured program that lasted for 7 months (August 2015 - February 2016). The structured peer-mentorship program ended earlier than anticipated from the study design due to protected time included in the M2 curriculum for dedicated USMLE STEP 1 studying. Consequently, M2s did not receive email prompts to contact their little sib during the last three months of the M1 academic year. The response rates for each survey administered can be found in Table 2.

**Figure 1.**
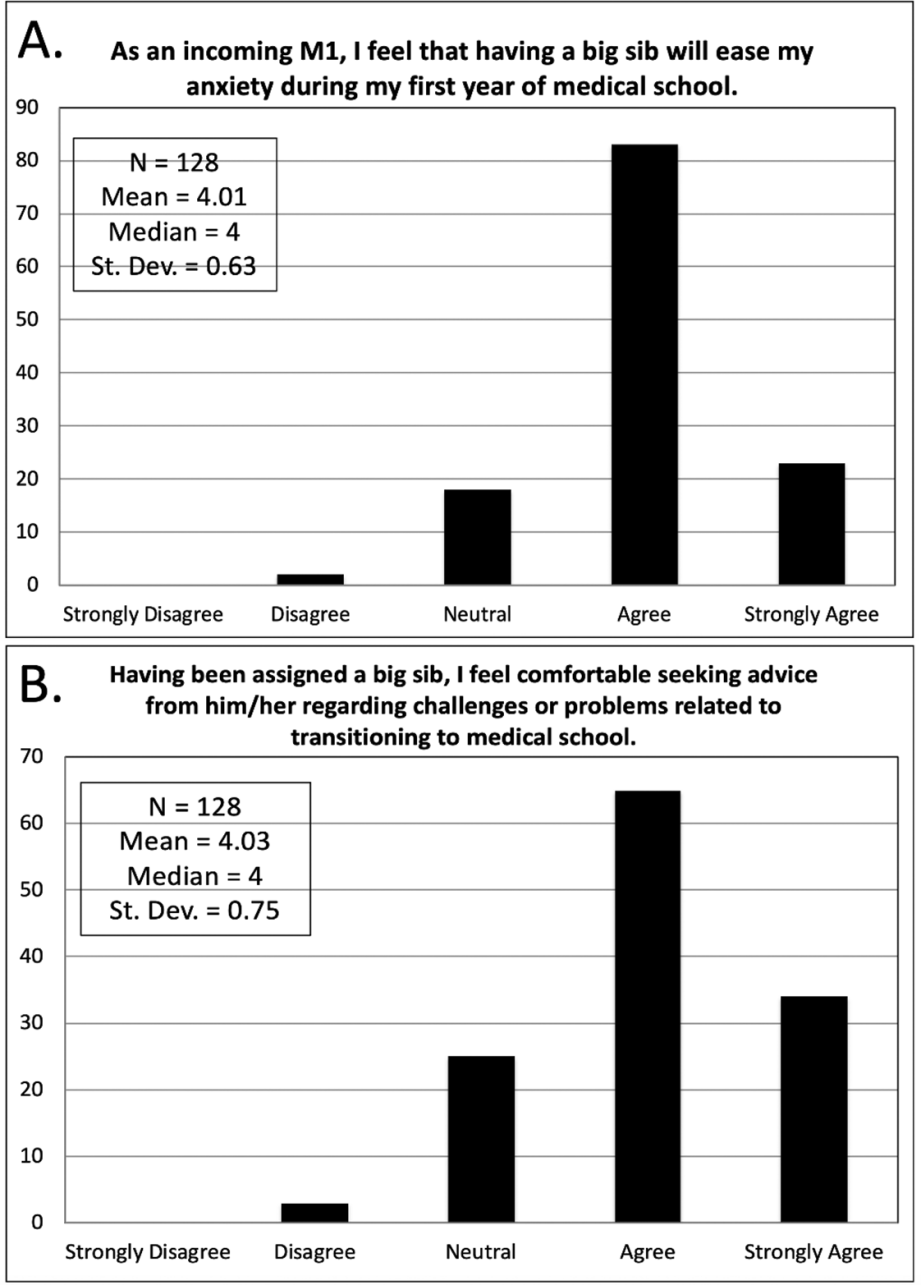
M1 Initial Assessment after M2 Pairing

M1s responded to a two-question survey prior to arrival on campus for matriculation. This survey focused on reduction of anxiety and comfort level seeking advice related to transitioning to medical school. This data is shown in Figure 1.

**Figure 2.**
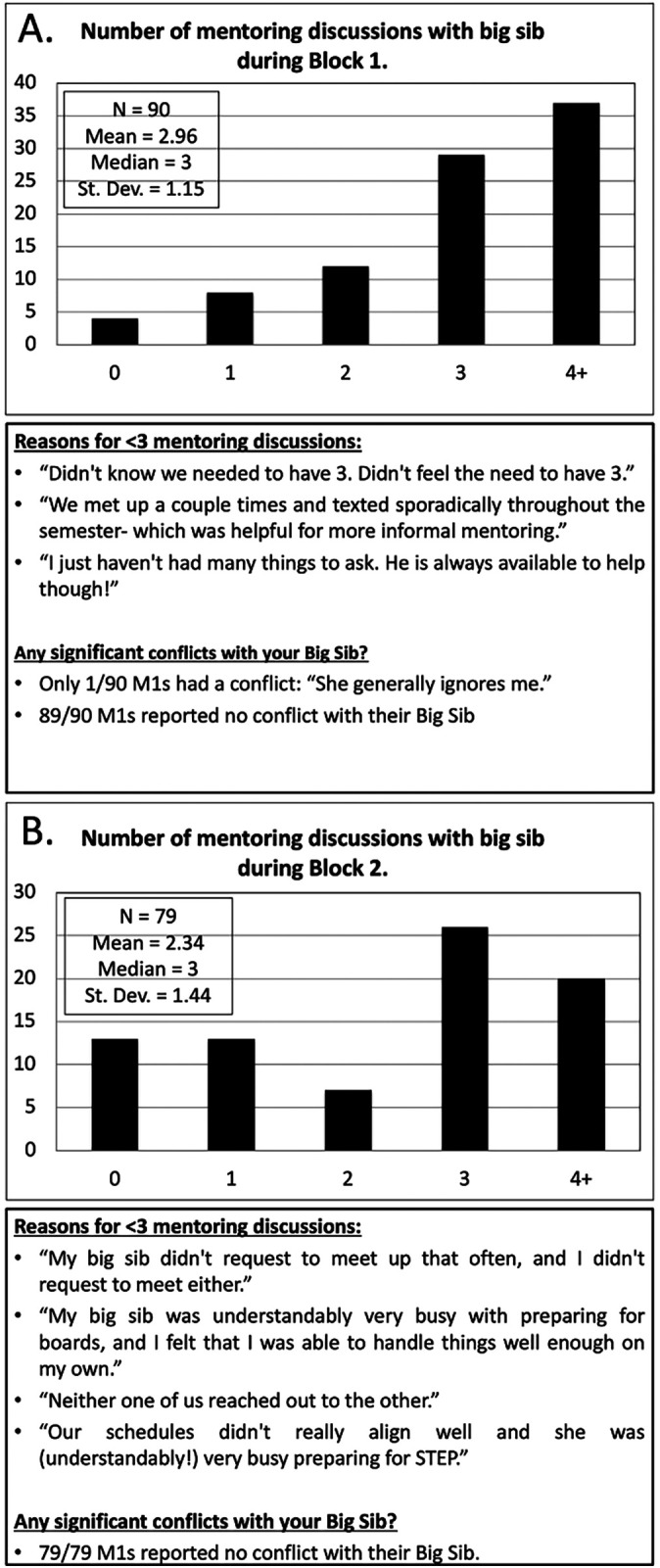
Block 1 and 2 Meeting Assessments

M1 responses were recorded based upon student agreement with (A) ease of anxiety and (B) development of comfort level in seeking advice from their assigned big sib. At the end of block 1, M1s responded to survey E, with over 33% (n = 37) of respondents reporting 4 or more mentoring discussions. The majority of M1s with less than 3 mentoring discussions responded that informal communication had replaced formal meetings or more than one mentoring session per module was unnecessary. Only one M1 reported significant conflict with their big sib. In block 2, a decreased number of M1s responded to survey E compared to block 1. A portion of M1s stated their big sib or themselves performed less outreach as one reason for less than 3 mentoring discussions. No sibling conflicts were reported during block 2. These results are presented in Figure 2.

**Figure 3.**
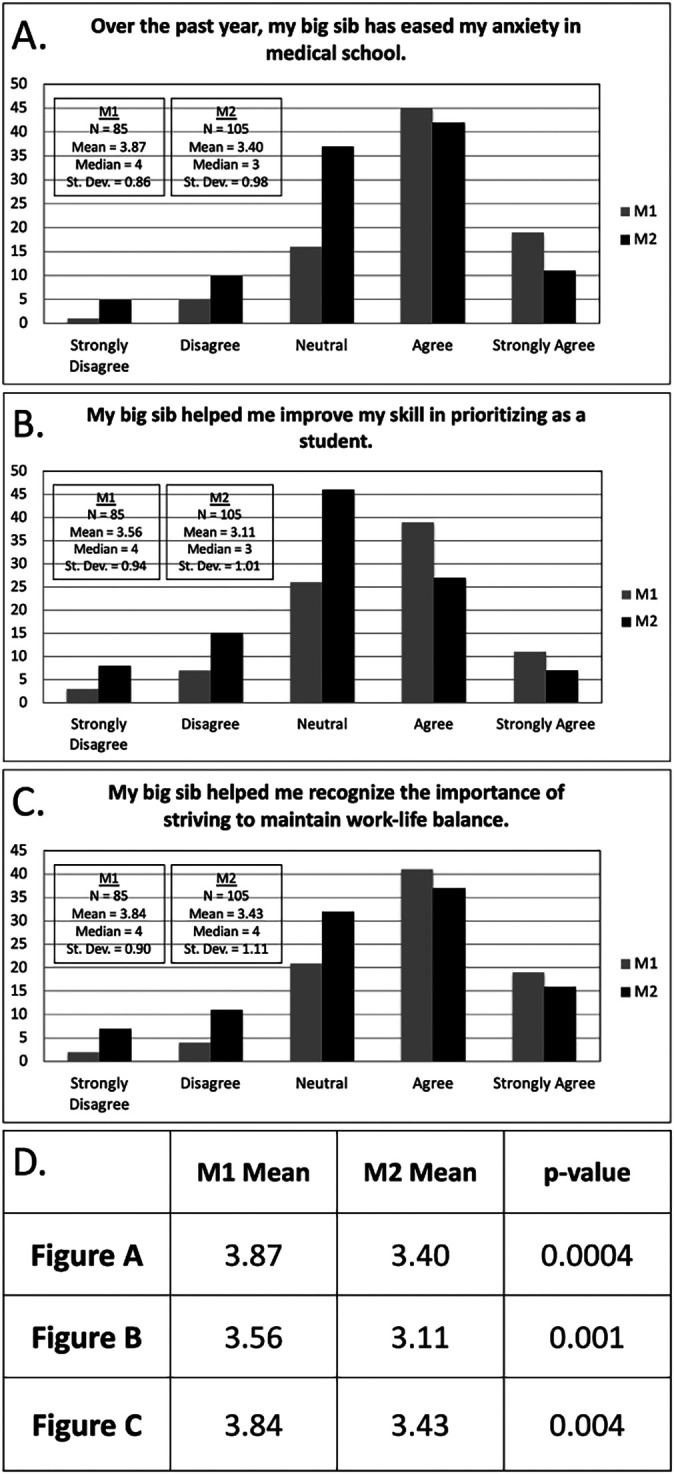
Comparison of Unstructured and Structured Peer Mentorship Programs

Figure 2 demonstrates M1s reported number of mentoring discussions, justifications for <3 mentoring discussions, and any significant conflicts with their big sibs during (A) block 1 and (B) block 2. Results from the M2 survey at the beginning of the academic year are found in Figure 3. M2s completed this survey at the start of their second year and M1s completed the same survey at the end of their first year. A comparison of M1 and M2 data in Figure 3 shows a statistically significant reduction in anxiety, improved prioritization skills, and increased awareness of the importance of striving to maintain work-life balance with *p* = 0.0004, 0.001, and 0.004, respectively, in favor of M1s utilizing a structured peer-mentorship program.

**Figure 4.**
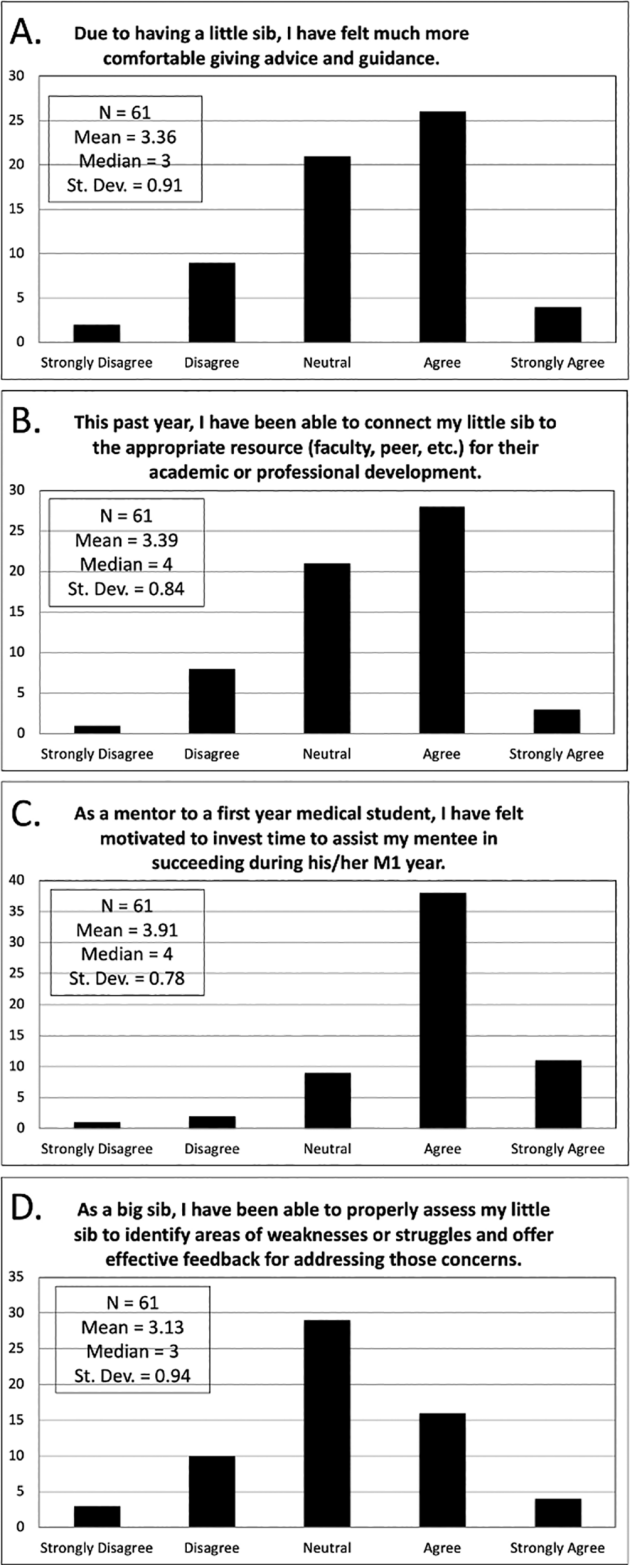
M2s Mentoring Skills Development

Figure 3 shows bar graphs of M1s (structured program) and M2s (unstructured) peer mentorship programs with respect to (A) reducing anxiety, (B) improved prioritization skills, and (C) striving to maintain work-life balance. Statistical comparison between structured and unstructured programs using α = 0.05 with an unpaired Student’s t-test demonstrated a statistically significant difference in all three groups in Figure 3D. At the end of block 2, M2s assessed their skills as mentors using a survey with the questions and responses shown in Figure 4. On average, the M2s felt largely neutral to agreeable in their ability to give advice, remain invested in their mentee’s success, and offer effective feedback as mentors in addition to identifying areas of weaknesses and providing resources for academic or professional development to their little sibs.

Figure 4 shows agreement of M2s in structured peer mentorship boosting (A) comfort level in giving advice and guidance, (B) referral ability, (C) investment effort, and (D) identification of weaknesses and challenges for their assigned little sib.

## Discussion

First-year medical students at our institution appear to have benefited from having a structured peer-mentoring program. Our data suggest that, compared to an unstructured peer-mentorship program, introducing guidance via talking points and prompting regular mentoring sessions significantly reduced anxiety, improved prioritization skills, and raised awareness of the importance of striving to maintain work-life balance-factors associated with a risk of burnout. To our knowledge, this study represents the first assessment of a structured peer-mentoring program aimed at reducing the burnout components in first-year medical students.

These benefits may be attributed, in part, to a program that prompted M2s to reach out to M1s at times of predictable stress during the academic year, such as preparing for their first objective structured clinical examination (OSCE) or written examination. Over the academic year, students reported having multiple mentoring discussions between siblings, especially early in the year. The reduction in mentoring discussions later in the year may suggest that students had adjusted to school and anxiety levels had declined from the start of medical school. Additionally, the observed decline in discussions could further be explained by the absence of the M2s for their protected time to prepare for USMLE STEP 1. However, evidence of unprompted discussions between siblings was noted in Figure 2B after only two months of structured mentoring in January and February. We would have expected on average 2 mentoring discussions across sibling pairs, but our data indicate a mean and median above 2, suggesting a persistence of sibling mentorship. This finding underscores the value of having a structured peer-mentorship program and students’ desire for continuity in peer-mentoring. Once established, recurrent mentoring discussions without formal guidance appear to be a sustainable outlet for mitigating burnout for first-year medical students.

Our results indicate that having a big sib reduced the anxiety of M1s. Interestingly, this benefit was notedeven beforestudents arrived at school or had met with their big sib. Additionally, having a big sib was associated with a willingness of M1s to seek advice about challenges related to transitioning to medical school. These observations emphasize the high levels of anxiety among incoming students and offer a readily available solution to alleviate it.

A surprising finding was that, throughout the academic year, only one M1 reported a poor relationship with his or her big sib. The outcome of this conflict remains unknown from our data. There may have been other sub-optimal sibling pairings that were not captured in our data due to a response rate of 55% and 48% for blocks 1 and 2, respectively. It is also possible that student concern over exposing conflicts between siblings for review by their peers and faculty may have inhibited additional reporting of poor relationships.

Finally, M2s also appeared to benefit from this program. Our data suggest that participating M2s were motivated to assist their mentees. For many M2s, this role may have been the first time they have functioned as mentors. This experience appears to have encouraged them to model constructive behavior, which may improve their performance later in the apprenticeship model of training (Stalmejjer, 2015; Ten Cate *et al.*, 2016; Merritt, Shah and Santen, 2017; Lyons *et al.*, 2017; Butler, Butler and Peabody, 2019; O’Conner, 2019; Konishi *et al.*, 2020). A majority of M2s felt neutral to positive in their ability to function as mentors. In particular, over 80% (n = 49) agreed or strongly agreed that participation in the program motivated them to invest time to assist their mentee during their M1 year as shown in Figure 4C. Participation in this program, therefore, appears to have augmented the professional development of many M2s by engaging them in a meaningful, longitudinal mentoring relationship. In contrast, only half of our M2s agreed or strongly agreed with their ability to give advice and guidance and connect students with appropriate resources, and only a third felt confident in identifying areas of weaknesses and offering effective feedback. These measures identify potential areas for improvement to better support M2s in their role as mentors through formal training.

There are several limitations to this study. First, data obtained was from a single institution, limiting the generalizability of our findings. Our data is further limited by the use of non-validated surveys in assessing self-reported anxiety, prioritization skills, and work-life balance understanding in contrast to objective measurements of these variables. Second, our data is limited to a single, truncated year. While our findings were promising, additional data is needed to further support our findings and its reproducibility. As part of this study, a declining response rate may represent selection bias leading to a non-representative sample of M1s and M2s. Third, we surveyed M2s about their experiences as little sibs at the start of their second year of medical school. This delay in surveying M2s after completing their first year of medical school introduces the potential for recall bias in the control group.

As this program evolves, we anticipate M1s who participated in a structured program may perform better as M2 mentors. These M2s may feel more confident when helping their little sibs address common challenges of the first year in medical school compared to M2s who had participated in an unstructured program. Subsequent iterations of the Sibs Program may also show even greater reductions in anxiety levels and improvements in prioritization skills and work-life balance. In addition, it would be helpful to collect data regarding the barriers to mentoring identified by M2s that could be used to develop strategies to improve their mentoring skills.

## Conclusion

Compared to an unstructured peer-mentoring program, a structured peer-mentoring program was associated with self-reported reductions in anxiety, improved prioritization skills, and greater awareness of the importance of striving for work-life balance among first-year medical students. These elements can ease the transition into medical school and are associated with reduced risk of burnout. Incoming medical students have positive associations with being assigned a peer mentor, and second-year medical students report that serving as a peer mentor motivated them to help mentees.

## Take Home Messages

• First-year medical students are at risk for burnout despite commonly available resources to help them manage stress.
• Compared to an unstructured peer-mentoring program between first- and second-year medical students, a structured program was associated with self-reported reductions in anxiety, improved prioritization skills, and greater awareness of the importance of striving for work-life balance among first-year medical students.
• Second-year medical students reported that serving as a peer-mentor motivated them to help their mentees and facilitated a sustained longitudinal mentorship.

## Notes On Contributors

**Gabriel A. Arenas** is an alumnus of the Northwestern University Feinberg School of Medicine, where he earned his medical doctorate in 2018. He is currently an obstetrics and gynecology resident at the University of Chicago Medical Center interested in medical education and mentorship.

**Gregory E. Brisson** is a clinical assistant professor of internal medicine at Northwestern Memorial Hospital. He is also the leader of the academic societies within the Northwestern University Feinberg School of Medicine.
