# Supplementary material for: The Sibs Program: A Structured Peer-Mentorship Program to Reduce Burnout for First-Year Medical Students
Source: MedEdPublish (2016). 2020 Dec 23;9:287. [Version 1] doi: 10.15694/mep.2020.000287.1 (PMC10697449; doi:10.15694/mep.2020.000287.1)
Supplement: Peer review history

# Reviewer report: Reviewer response for version 1

**DOI:** 10.21956/mep.20164.r31160

**Published:** 2020-12-28

**Authors:** BALAJI ARUMUGAM

**Affiliations:** 1 TAGORE MEDICAL COLLEGE AND HOSPITAL

This review has been migrated. The reviewer awarded 4 stars out of 5 Thanks for the opportunity to review this article. This is an educational intervention study compared the two groups who underwent un structured and structured peer mentorship program and ti was concluded that the first-year medical students appear to have benefited from having a structured peer-mentoring program which is evident statistically by unpaired t test between M1 and M2 students mean values. Two queries / suggestions to the authors1. Was there any interim analysis conducted or any pre and post comparison for M1 students done in this study?2. Complete Study protocol can be added as a supplementary file to get more clear picture on – the study population, recruitment, follow up, outcome surveys and analysis. Thanks regards Dr. A. Balaji

Reviewer Expertise:

NA

No decision status is available

# Reviewer report: Reviewer response for version 1

**DOI:** 10.21956/mep.20164.r31159

**Published:** 2020-12-23

**Authors:** Subha Ramani

**Affiliations:** 1 Harvard Medical School, Brigham and Women's Hospital

This review has been migrated. The reviewer awarded 4 stars out of 5 I enjoyed reading this paper about the big sib mentoring program at the Northwestern University medical school and agree with the authors that peer mentoring is an effective approach to enhancing wellbeing among junior medical students.The abstract summarizes the program and results well.The introduction provides a good lead in to why the program was designed and the need for this study. The problem is stated, the gap in literature mentioned and the goals of the program are reasonably clear.Under methods, the program has been described well. I really like the approach where the students are placed into societies and the sibling program constructed within the society. This provides many opportunities for engagement and society faculty availability for guidance. The talking points are also well done to ensure that the siblings have a framework for their conversations.Tables 1 and 2 provide informative details about the structure of the program and evaluation time points.The results section only consists of graphs and tables without a concise summary of what the most important results were. A lot of data have been collected and I am not entirely sure all of them are necessary to inform readers about the impact of the program. I could have been convinced of its effectiveness with less data. I would have actually liked to have seen some narrative or open-ended comments which would have been really meaningful.Overall the paper is well written, well referenced and would be of interest to all educators designing peer mentoring programs for undergraduate or postgraduate trainees.

Reviewer Expertise:

NA

No decision status is available
